# Cost-effectiveness of a pressure ulcer quality collaborative

**DOI:** 10.1186/1478-7547-8-11

**Published:** 2010-06-01

**Authors:** Peter Makai, Marc Koopmanschap, Roland Bal, Anna P Nieboer

**Affiliations:** 1 Department of Health Policy and Management, Erasmus University Rotterdam, the Netherlands

## Abstract

**Background:**

A quality improvement collaborative (QIC) in the Dutch long-term care sector (nursing homes, assisted living facilities, home care) used evidence-based prevention methods to reduce the incidence and prevalence of pressure ulcers (PUs). The collaborative consisted of a core team of experts and 25 organizational project teams. Our aim was to determine its cost-effectiveness from a healthcare perspective.

**Methods:**

We used a non-controlled pre-post design to establish the change in incidence and prevalence of PUs in 88 patients over the course of a year. Staff indexed data and prevention methods (activities, materials). Quality of life (Qol) weights were assigned to the PU states. We assessed the costs of activities and materials in the project. A Markov model was built based on effectiveness and cost data, complemented with a probabilistic sensitivity analysis. To illustrate the results of longer term, three scenarios were created in which change in incidence and prevalence measures were (1) not sustained, (2) partially sustained, and (3) completely sustained.

**Results:**

Incidence of PUs decreased from 15% to 4.5% for the 88 patients. Prevalence decreased from 38.6% to 22.7%. Average Quality of Life (Qol) of patients increased by 0.02 Quality Adjusted Life Years (QALY)s in two years; healthcare costs increased by €2000 per patient; the Incremental Cost-effectiveness Ratio (ICER) was between 78,500 and 131,000 depending on whether the changes in incidence and prevalence of PU were sustained.

**Conclusions:**

During the QIC PU incidence and prevalence significantly declined. When compared to standard PU care, the QIC was probably more costly and more effective in the short run, but its long-term cost-effectiveness is questionable. The QIC can only be cost-effective if the changes in incidence and prevalence of PU are sustained.

## Background

A pressure ulcer (PU) is a preventable condition that affects patients with impaired mobility, especially the elderly [1]. PUs are classified from grades 1 to 4, or least to most severe. The average prevalence of PUs in the Netherlands is 7.9% in assisted living homes and 18.3% in nursing homes [2]. Incidence varies between 2.9% and 4.5% in intensive care [3]. No incidence data are available for the Dutch long-term care sector. The probability of healing within 90 days varies with severity: 67% (grade 2), 44% (grade 3) and 32% (grade 4) [4]. PUs can interfere with recovery, cause pain and infection [1], and increase mortality (OR = 1.4 after adjusting for risk factors) [5]. According to a study by Franks [6] the quality of life of PU patients is no worse than the general population of nursing home patients; a study by Fleurence, [7] however, claims that PUs decrease quality of life. The treatment of PUs costs between € 89 million and 1.9 billion, or 0.1% to 1% of total Dutch healthcare costs [8,9]. Because they are preventable, it is safe to say that PUs should not occur in the first place.

Preventable conditions requiring a common and perhaps demanding treatment like PUs are likely candidates for Quality Improvement Collaboratives (QICs), [10,11], in which different healthcare organizations address a certain problem by implementing specific solutions and sharing the results [12]. A QIC program team includes experts in both the health condition and methods of quality improvement. According to a recent systematic review, QICs have shown moderate effectiveness in terms of patient outcomes [10] and several studies suggest effectiveness of QICs for PUs in particular [13,14]. Despite the popularity of QIC's, the cost-effectiveness of QICs is rarely considered [10], in fact only a study by Huang addressed this aspect [15].

This is not surprising, since the costs of quality improvement projects are not well established, and organizations generally do not or cannot assess the benefits of participation [16]. There are currently no published studies on the cost-effectiveness of a PU QIC in particular. Several studies have been published on the cost-effectiveness of the materials for PU treatment and prevention [7,17-19], and the one study we found that focused on labor costs [20] considered only nurse staffing time and disregarded preventive activities. We did identify a cost- effectiveness study on a PU quality improvement project [21], but it did not involve a QIC. This study adds to the literature by giving a detailed account of the PU sub-program of the "Care for Better" QIC, a Dutch healthcare collaborative[22]. The aim of this article is to answer the question: Was this PU QIC cost-effective when compared to standard PU care?

## Methods

### Design

Our study was conducted from a healthcare perspective, considering both direct costs of PU care and costs of the QIC for a period of one year. A prospective pre-post design was used with one-month measurement periods to collect data on costs and effectiveness. We established cost effectiveness by comparing data at the end of the project year to standard care (i.e., the state of the sample before the QIC intervention). We built a Markov model to establish standard care (i.e. simulate a control group), and to determine the effect of the collaborative after a year. To extrapolate results to one additional year, we have expanded this model. Probabilistic sensitivity analysis was applied to treat uncertainty in the model parameters. QALYs and ICERs were calculated for a two year period (project year and extrapolated year).

### Setting

The Care for Better QIC operates in the Dutch long term care sector (nursing homes, residential care homes, and home care). This study is limited to nursing and residential homes. Patients are not admitted with PU as a main condition, but have underlying chronic conditions affecting their daily functioning. The nursing home patients typically stay in the facilities for two to three [23,24] years until death, and are seldom discharged.

### Description of the Collaborative

The overall goal of the Care for Better PU QIC was to reduce the prevalence and incidence of PUs by 50% in 25 participating organizations over the course of a year by increasing evidence-based preventive measures and decreasing non-useful preventive measures (table 1) [1], thereby reducing the need for treating PUs. The project was implemented in three consecutive rounds because not all 25 organizations could be accommodated by the Care for Better PU QIC at one time.

**Table 1:** Patient characteristics, outcomes and changes in process

	*Non-selected patients*	*Selected patients*	
Number of patients	254	88	

BMI (average)	26	26 (5)	

Age (average)	80	82	

Females (average)	169 (67%) F	60 (68%) F	

Patients at risk of pressure ulcers (average)	254 (100%)	88 (100%)	

***Comparison of clinical effects***	*Baseline*	*Baseline*	*After*

*Prevalence*			
Grade 1	50 (20%)	21 (23.9%)	16 (18.2%)
Grade 2	9 (3.5%)	10 (11.4%)	2 (2.3%)
Grade 3	3 (1.2%)	1 (1.1%)	1 (1.1%)
Grade 4	5 (2%)	2 (2.3%)	1 (1.1%)
Total	59 (27%)	34 (38.6%)	20 (22.7%)*

*Incidence (1 month)*			
Grade 1	19 (7%)	10 (14.7%)	4 (4.5%)
Grade 2	6 (3%)	3 (3.4%)	0 (0%)
Total	25 (9%)	13 (15%)	4 (4.5%)*

*Useful interventions*			

Risk assessment	254 (100%)	88 (100%)	88 (100%)

Using a 30-degree side to side turn at least every 4 hours	24 (9%)	7 (8%)	9 (10%)

Preventive mattress	78 (30%)	24 (27%)	40 (45%)**

Involving patients in prevention	41 (16%)	3 (3%)	7 (8%)

Involving family/friends/caregivers in prevention	26 (10%)	3 (3%)	9 (11%)

Reactivation and mobilization by paramedics	10 (4%)	3 (3%)	11 (13%)

Smearing of the skin in case of incontinence	30 (11%)	8 (9%)	9 (11%)

Assessing nutritional state and preventing nutritional deficiency	13 (5%)	12 (14%)	4 (5%)

Inserting a catheter to prevent maceration of the skin	3 (1%)	1 (1%)	1 (1%)

Ensuring a clean, dry and square lower layer of bedclothes	52 (20%)	8 (9%)	12 (14%)

*Non-useful interventions*			
Smearing the skin (with topical agents) to prevent disturbance in blood supply caused by pressure	50 (20%)	23 (26%)	6 (7%)*

Massage	3 (4%)	0 (0%)	2 (2%)

Using a 90-degree side to side turn at least every 4 hours	2 (1%)	0 (0%)	3 (4%)

*P < 0.05

**p < 0.005

**Figure 1:**
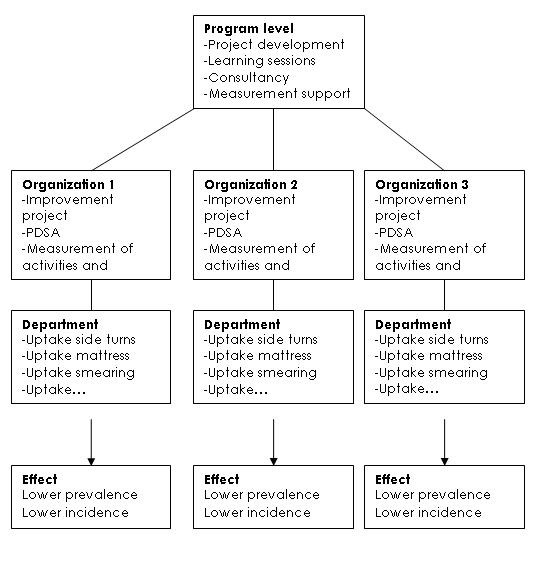
**The structure of the collaborative**.

The Care for Better PU QIC carried out activities on three intertwining levels: program, organizational, and departmental (figure 1). The program level consisted of a core team of experts who guided the organizations' project teams, defined the collaborative's goals, and organized three "learning sessions" during the year at which project teams could be taught about quality improvement methods and preventive nursing measures, and share their results with the other teams. Between the learning sessions, the core team of experts provided project teams with coaching.

The participating organizations formed project teams who attended the learning sessions and were the effective drivers of the implementation in pilot departments of the organizations. Project teams had considerable freedom in the type of preventive nursing measures implemented and how they were applied, but were encouraged by the experts to formulate SMART (Specific Measureable Attainable Realistic Timely) goals and to work with PDSA (Plan Do Study Act) cycles between the learning sessions. The PDSA cycles began with "action plans" followed by introducing new interventions at the departmental level. Periodic measurement of results were documented. At the end of the cycle, the new interventions were meant to be used in the entire organization, and meant to be incorporated into the work of professionals. In this manner, successful teams standardized the new interventions and made changes permanent. In addition it was expected from the teams that they learn methods of continuous quality improvement, in other words teams were meant to continue working with the PDSA cycle after the QIC program was finished.

During the one-month measurement periods preceding the learning sessions, project teams registered 18 different preventive measures carried out by caregivers, as well as the prevalence, incidence and severity of the PUs. These registrations consisted of 12 measurement moments, measuring every patient on the pilot department every two to three days. The first measurement was conducted end October to end November 2006 or from beginning of November to the beginning of December depending on the institution. The intermittent measurement period was in June, and the last measurement period was in November 2007. The measurements were organized by the Dutch National Expertise Center for Nursing and Caring, and were carried out by the project teams themselves.

### Case-selection and study population

To capture possible learning effects over the course of the year, data was used from the third round. A total of seven departments in three different organizations were investigated in detail. The following criteria were used to select cases:

1. Data was available for both first and last measurement period.

2. At least one department had a low initial PU prevalence, at least one department had an average PU prevalence, and at least one department had a high PU prevalence.

**Figure 2:**
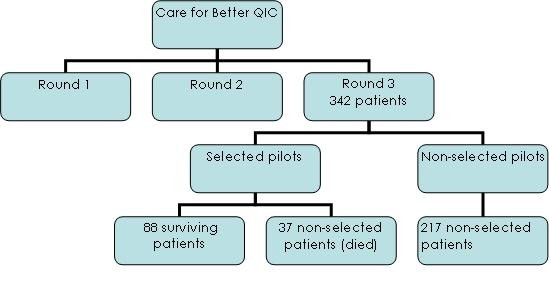
**Selection process of the 88 patients**.

Using this criteria, 88 patients were selected - ranging from 9-19 per department - to determine cost-effectiveness (figure 2). Their characteristics compared to the non-selected cases in the third round are described in table 1. To determine the representativeness of the selected cases vis-à-vis the entire patient population, we compared the 88 patients' risk for PUs, age, sex, and BMI to the non-included patients in round three of the project using ANOVA at baseline.

### Determination of effectiveness

We used effectiveness data on the prevalence and incidence of PUs collected by the organizational project teams. Prevalence was computed by averaging the number of patients with PU divided by 88 over the whole measurement month. Incidence was computed as the number of new PU cases during the measurement month divided by 88. To determine effectiveness, we compared the before- and after-project PU prevalence and incidence of the 88 patients using a t-test.

### Assessment of costs

Cost data associated with the project and the prevention and treatment of PUs were collected for the central activities on the program level, the project activities within the organizations, and the individual treatment of patients (departmental level). Identification and valuation of costs are displayed in table 2.

**Table 2:** Activities of caregivers and treatment material used

*Program level*			*Amount*
Labor	Program activities (project design, expert meetings, recruitment, organizing working conferences, mid-term report, final report etc.)	Program experts	4696 hours
	
	Program support	Program experts	635 hours
	
	Knowledge management (publications, etc)	Program experts	175 hours

Materials			Lump sum

Other costs			Lump sum

***Organizational level***			***Average***

Labor	Project activities (coordinating the project, writing action plans, reports, etc.)	Project leader	8 hours (per week)
	
	Clinical level project implementation	Project member	2 hours (per week)
	
	Learning session participation	- Project leader - 2 Project members	76 hours (total each)
	
	Staff knowledge testing	- Nurses - Caregivers	30 min (total each)
	
	Caregiver training	- Specialized nurse - Caregivers	3.5 hours (total each)
	
	Specialist training	Nurses	8 hours (total each)
	
	Project meetings	- Project member - Nurses - Caregivers	8 hours (total each)

	Measurements	Nurses	1 hour (per month)

***Departmental level***			***Average/day/patient***

Useful interventions	Risk assessment	Nurses	10 sec
	
	30-degree side turn at least every 4 hours	Caregivers	35 min
	
	Involving patients in prevention	Nurses	2 sec
	
	Involving family/friends/caregivers in prevention	Nurses	0.4 sec
	
	Reactivation and mobilization by paramedics	Paramedics	4 min
	
	Smearing the skin with topical agents in case of incontinence	Caregivers	2 min
	
	Assessing nutritional state and preventing nutritional deficiency	Caregivers	4 min
	
	Inserting a catheter to prevent maceration of the skin	Caregivers	3 min

Non-useful interventions	Ensuring a clean, dry and square lower layer of bedclothes	Caregivers	7 min
	
	Smearing the skin (with topical agents) to prevent disturbance in blood supply caused by pressure	Caregivers	2 min
	Massage	Caregivers	1 min
	
	90-degree side turn at least every 4 hours	Caregivers	30 min
	
	Usual treatment grades 1-2	Caregivers	7 min
	
	Usual treatment grades 3-4	Caregiver	15 min

Materials		**Type**	**Number/patient**
	
	Basic mattress	Start	1
	
	Mattress (grades 1-2)	SLK 1	1
	
	Mattress (grades 1-2)	Dionica	1
	
	*Mattress (grades 3-4)*	SLK 2	1
	
	Mattress (grades 3-4)	Duo-care	1
	
	Mattress (grades 3-4)	Quatro-care	1
	
	Pillow (prevention)	Foam pillow	1
	
	*Pillow (grades 1-2)*	Normal PU pillow	1
	
	Pillow (grades 3-4)	ROHO	1

#### Program and organizational

Program costs were obtained from the central project budget. Items included expected project time, lump sums for materials, and miscellaneous costs. To ascertain organizational level costs, the organizations' project leaders supplied us with detailed plans and reports. They also furnished the individual amounts of time invested in the project by the teams and other employees for various activities (training, participation in learning sessions, writing plans, project implementation). To establish the project costs, we multiplied the number of hours spent on the project by the average hourly wages of the project team members.

#### Departmental

We used project documentation to identify the before- and after-project differences in PU preventive measures and the number of mattresses and pillows used. The type of mattresses and pillows were taken from the organizations' treatment protocols; their rental rates were collected from the suppliers of the organizations (table 3). Since other materials used for PU care (creams, dressings, and the like) were not reliably administered, we assumed they did not change during the project. Studies have also shown these costs to be marginal compared to the total cost of care [9]. We also didn't account for changes in organizational overhead costs, because the changes all took place in the departments themselves, and had no effect on other parts of the organizations. Time spent by staff on activities related to preventive care was collected through interviews with project members, who were asked to give an average, minimum, and maximum value for each preventive measure. In the context of an average long-term care stay of 2.8 years [25], with 66% remaining until death [26], we assumed that PUs do not cause extra days of care. We computed the cost of personnel at the departmental level by multiplying the time spent on PU care by the hourly wage of caregivers in the organizations. We used the wage schedule of the 2006 collective agreement of Dutch nursing home employees [27].

**Table 3:** Wages of staff and prices of materials

*Labor*		
	Program experts	115.00

Project (hourly)	Project leader	36.82
	
	Project member	31.56
	
Departmental (hourly)	Paramedic	31.56

	Dietician	31.56
	
	Specialized nurse	34.71
	
	Nurse	31.56
	
	Other caregiver	18.94

***Materials***		

Project (totals)	Project materials	50,000
	
	Other collaborative costs	64,000

Departmental (daily rental price)	Basic mattress	1.11
	
	SLK 1 (grades 1-2)	2.56
	
	Dionica mattress (grades 1-2)	0.64
	
	SLK 2 (grades 3 & 4)	4.52
	
	Duo-care mattress (grades 3-4)	3.29
	
	Quatro-care mattress (grades 3-4)	13.15
	
	Foam pillow	0.03
	
	Normal PU pillow	0.04
	
	Special PU pillow (ROHO)	0.18

To compute an overall cost per patient value, the cost of the collaborative was evenly allocated to the participating project teams. Organizational level costs were evenly allocated to the patients. Average daily costs were computed per patient per disease state and converted into monthly values.

### Decision Analytical Model

To determine the effect of the collaborative compared to standard care after a full year, we have built a decision-analytical model (Markov model) based on our data from the collaborative to simulate standard care (i.e. control group). In building the model we have used the method outlined by [28]. The model had health states consisting of no PU, single PUs grades 1-4, and multiple PUs grades 1-4. For the first year (when the collaborative ran), we used two sets of transition probabilities: one for the simulated control-group, and one for the intervention group. To establish standard care, we converted incidence and PU healing during the first measurement month into monthly transition probabilities, giving a simulation under the assumption there was no collaborative. With the intervention group we based transition probabilities on the events of the first year (based on the data from the first and last measurement month) and we transformed these yearly transition probabilities into monthly transition probabilities. This monthly modeling was necessary to give a more precise change in effects and costs over this first year, and to make the two simulations comparable. Both arms of the model were run 12 times to simulate a one-year program.

To extrapolate the results for an additional year, we also included mortality in the model by introducing a death state into the model, and using the average mortality of nursing home patients in the Netherlands [29] as a transition probability. The simulated control-group thus consisted of no PU, single PUs grades 1-4, and multiple PUs grades 1-4 and death, with the transition probabilities adjusted accordingly. The intervention group, - in addition to a death state - three scenarios were created: total sustainability, partial sustainability and no sustainability. In the total sustainability scenario, we have assumed that the process has the same dynamic as during the first year. In the middle scenario, we have assumed that the dynamic is broken, but the new measures are sustained, as well as the achieved results. In the no sustainability scenario, we assumed that the improvement is slowly reversed, therefore we have used the inverse transition matrix of the first year.

In order to get an idea if such a collaborative are worth financing, it is important to place it in the context of a policy decision environment, to allow a tradeoff between costs and QUALY-s. Quality of life (Qol) weights for PU patients and for the general geriatric population were obtained from the literature. The Qol weight was 0.703 for pressure-ulcer free nursing home patients, 0.68 for those with single PUs of grades 1 and 2; 0.5 for multiple PUs of grades 1 and 2; and 0.36 for severe PUs (grades 3 and 4) [7,24,30]. Cost data were the costs collected from the collaborative.

To establish the effect of the uncertainty in the parameters of the base case we conducted a probabilistic sensitivity analysis, assuming a lognormal distribution for costs and effects. A Monte Carlo simulation was run with 10,000 iterations per scenario.

We used standard discount rates recommended by the Dutch guideline for pharmaeconomic studies (4% for costs 1.5% for effects) [31].

## Results

### Patient characteristics

The 88 selected patients were not significantly different in age, sex, or BMI from the non-selected patients participating in the third round of the project. This was true for baseline and terminal measurement points.

### Effectiveness

As can be seen in table 1, the prevalence and incidence of PUs in the selected patient group is lower after the collaborative, primarily due to reduction of less serious ulcers (grades 1 and 2). The participating patient group also had a lower prevalence and incidence of PUs compared to the non-participating patients. The uptake of useful interventions generally increased or did not change significantly over time. We also observed the uptake of non-useful interventions.

### Costs

Table 2 shows a breakdown of materials used and time spent on activities by all participants. The most time-consuming activity was intermittently turning the patient to the side. Materials and time are translated into costs in table 3. The program experts have the highest hourly wage, the caregivers the lowest. The daily rental price of mattresses varies substantially.

Table 4 shows that the project created a savings in variable nursing costs while increasing costs of preventing and treating PUs. Most of the cost goes to personnel, followed by mattress rental. Costs fluctuated primarily by the reduction of grades 1 and 2 PUs, since the number of severe ulcers did not change. In addition, the one-year project costs for the organizations were larger than the possible savings of a reduction of PUs. Therefore, the initial investment can only be recovered over a longer time period.

**Table 4:** Costs per person treated for selected patients

			PU Grade									Average costs	
**STANDARD CARE**			0	1		2		3		4			
					
			n/a	single	multiple	single	multiple	single	multiple	single	multiple	monthly	yearly
	
	Pre-vention	Labor Mattress Pillows	11.02 2.22 0.00	13.00 19.18 0.18	23.29 28.53 0.32	148.43 200.01 0.43	47.62 41.57 0.63	n/a	132.55 394.52 0.00	n/a	279.82 232.21 3.05		
	
	Treatment		0.00	68.38	64.82	59.17	68.64	n/a	142.01		118.34		
	
	*Total standard care costs*		*13.15*	*100.73*	*114.57*	*408.04*	*115.89*	*n/a*	*669.09*	*n/a*	*657.10*	*84*	*1026*

**QIC**	Pre-vention	Labor Mattress Pillows	30.80 7.38 0.34	45.86 42.37 0.45	110.25 49.57 0.51	123.30 47.95 0.41	n/a	n/a	192.60 82.19 0.00	278.10 98.63 1.32	n/a		
	
	Treatment		0.00	59.17	66.27	56.81	n/a	n/a	142.01	142.01	n/a		
	
	*Total QIC clinical costs*		*38.52*	*147.86*	*226.86*	*228.46*	*n/a*	*n/a*	*416.71*	*520.10*	*n/a*	*79*	*969*
	
	Program costs												323
	
	Organizational costs												1550
	
	*Total QIC costs*												*2842*

### Modeling and sensitivity analysis

**Figure 3:**
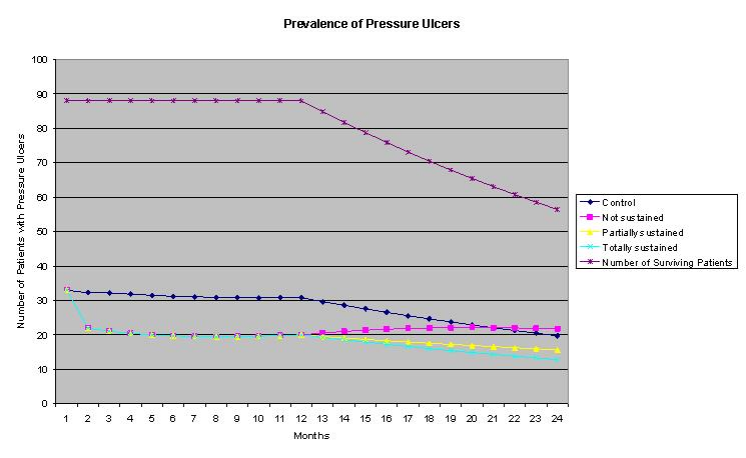
**Number of patients with pressure ulcers for two years after the start of the QIC**.

The prevalence of PUs over the course of the extrapolated year depends on whether or not the change in incidence and prevalence are sustained (figure 3). If changes are not sustained at all, any success realized during the year in terms of prevalence is lost. If changes are partially sustained, prevalence slightly increases in the second year; in the scenario where changes are fully sustained, prevalence remains low.

From a healthcare perspective, the costs of PU care increased as a result of the project. At the same time, the project raised the average Qol of patients. Although the exact value of the QALY is debatable, there is a Dutch policy advice [32] stating that the values should be maximally €80,000 for patients with high disease severity. The QIC's incremental cost-effectiveness ratio after two years is above this limit of 80,000 €/QALY except for the most optimistic scenario where changes are completely sustained (table 5).

**Table 5:** Incremental costs, quality of life and cost-effectiveness ratio

	Not sustained	Partially sustained	Totally sustained
Difference in cost per person	€ 2.208 Probability = 0.97	€ 2.072 Probability = 0.97	€ 2.037 Probability = 0.97

Difference in Qol per person	0.016820965 Probability = 0.74	0.023361 Probability = 0.74	0.02594592 Probability = 0.75

ICER	131 253	88 692	78 517

**Figure 4:**
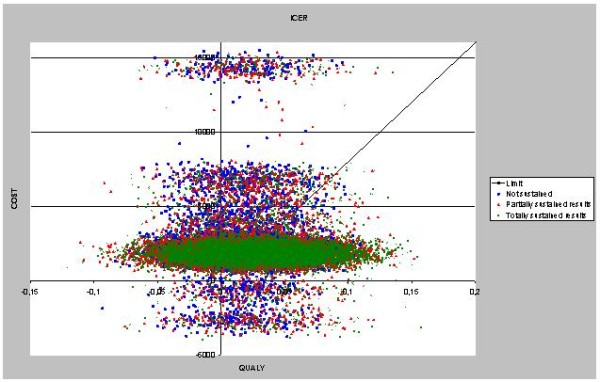
**Incremental costs and effects from Monte-Carlo simulation for three sustainability scenarios**.

The sensitivity analysis (figure 4) allows us to investigate the robustness of our results. The joint probability of the ICER being below 80,000 along with a positive effect on Qol is 37% for the not sustained scenario, 47% for the partially sustained scenario, and 50% for the totally sustained scenario. Therefore there is no clear indication of the collaborative being effective after two years, and there is a high probability that it is more costly in every scenario.

## Discussion

### Summary of main results

The QIC significantly reduced the PU prevalence when the measurements before and after the collaborative are compared. This decrease was mainly due to the decrease of non-severe PUs (grades 1 and 2). The Qol of patients probably did not increase significantly.

Even though the variable costs of the organizations decreased, the large project costs of the QIC increased healthcare costs overall. Therefore, a QIC can only be cost-effective if the efforts to reduce PUs are sustained. In other words, short-term effectiveness is a necessary, but not a sufficient condition for long-term cost-effectiveness.

### Sensitivity of the results

The sensitivity analysis showed considerable uncertainty in the results of the model and thus it is not possible to indicate clearly that the intervention was cost-effective. The uncertainty lies in the effects of the collaborative; it is only moderately probable that the patient's quality of life will increase. This may be caused by the fact that the difference in quality of life of a regular nursing home patient and a PU patient (independent of severity) is very small [6], which makes detection of change difficult. In this study, the difference in Qol between a patient without a PU and a patient with a low-grade PU was minimal.

It is likely that the intervention is more costly than standard PU care; this study, however, works with a different assumption than previous studies, therefore the savings reached by preventing PUs are lower than that which can be found in the literature [9]. This study assumed that PUs in the long term care sector do not cause extra patient days because 66% of nursing home patients receive long-term care [26] or die as in-patients. Therefore, we considered only the costs associated with PUs and their prevention. This is contrary to a previous Dutch study [9] that assumed PUs caused additional patient days in the long term care sector.

### Limitations and Strength

The main limitation of this study is that it was based on an observational study. This limitation has far-reaching consequences. Because of the lack of case-mix measures for the population, we were only able to include the small number of cases that survived the duration of the study, while ignoring cases that died during the study. In addition, overrepresentation may be a problem because we worked with self-reported data. Therefore we cannot say with certainty that the selected cases were representative of the whole population. Furthermore the results are prone to the biases of any observational study, namely, secular trends; therefore it is not certain that this decline actually happened because of the collaborative. It should be noted that secular trends were far slower then the improvement in the selected patients: according to the LPZ panel data from 2006 and 2007[33,34], the prevalence of pressure ulcers decreased from 24% to 18.3% in Dutch nursing homes and from 11% to 7.9% in assisted living facilities. Therefore it is not plausible that the decline in PU-s in the collaborative was caused exclusively by secular trends. Besides secular trends, selection of the cases may have had an effect on the precise cost per patient ratio. First including the costs of the remaining teams (9 successful and 6 unsuccessful teams) would have slightly increased the central cost per collaborative per patient. Second the project costs made by unsuccessful teams would slightly raise the average project cost, but since these teams did not complete the project these costs are small in comparison to the costs made by the successful departments. Therefore large biases are unlikely in the average cost/patient ratio.

Caution is called for when interpreting the long term effects of a collaborative. On one hand the small number of cases made the decision-analytic modeling difficult because the probabilities of incidence and healing in the model may not be representative for the whole group. On the other hand there is the question of which sustainability scenario is most realistic. There is scarce evidence in the literature about sustaining the changes of a QIC when the project is over [10], raising the question of whether a collaborative would ever be cost-effective. Even in organizations where the results are sustained for an additional year, the question of how far in the future the changes can be sustained remains. This is especially important because sustaining the changes is a prerequisite for the organizations participating in the QIC to regain the initial investment. The PU QIC involved staff training, and the significant rate of labor fluctuation characteristic of Dutch caregivers (10% annually) [35] may endanger sustainability in the long run.

The major strength of this study is that it is one of the first attempts to address the cost-effectiveness of a PU QIC. This study gives detailed information on the costs on the program level, the project costs within the organizations, as well as the differences in the costs of nursing activities. In addition we have put serious effort into decreasing the effect of design limitations. By simulating a control-group based on the real data of first measurement month we could visualize a situation where no attention would have been paid to PU-s, a situation in which all conditions are held the same. In other words we have been able to control for every variable except for changes caused by secular trends. Since control-groups are usually not feasible for QICs, simulating control-groups may be a feasible and promising approach to evaluate their cost-effectiveness, naturally with this limitation in mind.

Additional research using an appropriate-case-mix adjustment is needed to determine the effects of a PU QIC and to establish incidence and healing rates in a larger sample that includes the home care sector. Furthermore, additional research is needed on the effects of PU collaboratives using cluster-randomization and Qol measurements sensitive enough to detect changes in nursing home patients. Finally, the long term effects are also worthy of investigation, focusing especially on effective methods for sustaining beneficial changes.

## Conclusions

During the PU QIC the incidence and prevalence of PUs significantly declined thus reducing variable costs of organizations and probably realized small gains in quality of life. From a healthcare perspective, the collaborative was probably more costly and more effective in the short run than standard PU care. Long term effects are highly sensitive to the sustainability of the changes in nursing method. Running a collaborative costs money and profitability depends on the extent to which teams manage and sustain new working methods. Further research is needed to know how the improvement cycle plays out over a longer time period.

## Competing interests

The authors declare that they have no competing interests.

## Authors' contributions

PM has acquired and analyzed the data, drafted the manuscript and approved the final version. MK has made substantial contribution to the interpretation of the data, critically revised the manuscript for important intellectual content and approved the final version. RB revised the manuscript and approved the final version. AN has contributed to the study design, critically revised the manuscript for important intellectual content, and approved the final version.
